# Tobacco Use Among Adolescent Students and the Influence of Role Models

**DOI:** 10.4103/0970-0218.66891

**Published:** 2010-04

**Authors:** Rahul Sharma, Vijay L Grover, Sanjay Chaturvedi

**Affiliations:** Department of Community Medicine, UCMS and GTB Hospital, New Delhi, India

**Keywords:** Adolescent, behavior, students, smoking, tobacco use, role models

## Abstract

**Background:**

Seventy per cent of premature deaths among adults are due to behavioral patterns that emerge in adolescence, including smoking.

**Objective:**

The objective was to study the prevalence of tobacco use among adolescent students in South Delhi and its epidemiological correlates.

**Materials and Methods:**

This was a cross-sectional study.

**Setting:**

Three schools and two colleges of South Delhi were chosen. There were 550 adolescent students aged 14-19.

**Statistical Analysis:**

Statistical analysis was done using proportions, the chi-square test, and multivariate logistic regression.

**Results:**

A total of 88 (16.0%) students reported having ever tried cigarette or bidi smoking. The prevalence of current smoking was 7.1%. Exactly 10% (55) of the students reported having ever used smokeless forms of tobacco. The prevalence of tobacco use overall was found to be 20.9%, and was significantly higher (P=0.016) among the males than the females. Tobacco use was found to be significantly associated with having seen a brother/sister smoke (OR 5.15), best friend smoke (OR 2.92), and belonging to a nuclear family (OR 1.96).

**Conclusions:**

Tobacco use is still an important risk behavior among adolescent students. This study found a strong association of tobacco use by the adolescents with their having seen various role models ever smoking.

## Introduction

The World Health Organization (WHO) has defined ‘adolescents’ as persons in the 10 to 19 years age group.(1) People in the age group of 10-19 years, comprise 22% of the Indian population.(2) The World Health Organization estimates that 70% of premature deaths among adults are due to behavioral patterns that emerge in adolescence, including smoking, violence, and sexual behavior.(3)

The present study sought to cover six categories of the important health risk behaviors among adolescents. These included tobacco use, alcohol and other drug use, sexual risk behaviors, unhealthy dietary behaviors, inadequate physical activity, and behaviors that may result in injuries and violence. WHO mentions that many of the leading causes of premature death, disease, and disability in present and future can be reduced by preventing these six categories of interrelated risk behaviors.(4)

Adolescence is generally divided into three stages of development: early (10-13 years), middle (14-15 years), and late adolescence (16-19 years) stages.(1,5) It is usually seen that risk-taking behaviors begin to manifest from the middle adolescence onward.(6) Hence, the present study focused on the middle and late stages of adolescence. The subjects were thus chosen from the corresponding age group of 14-19 years.

It is seen that smoking and drinking become symbols of maturity and independence, among the young people. For them, the use of tobacco provides an opportunity for taking part in a behavior that defies established social norms.(7) Boys are more likely than girls to smoke, drink, and use drugs. This holds true in developing countries too, although rates for girls are increasing faster.(3) The behaviors of the important people in the immediate environment of the adolescents whom they may look up to as role models are likely to influence the activities of the adolescents. In this paper, the findings related to the tobacco use behavior among the adolescent students are presented.

## Materials and Methods

The study was a cross-sectional analysis of the subject population. The units of the study were 14 to 19 years old adolescents studying in various schools and colleges in South Delhi. The study being a doctoral thesis research was reviewed and approved by the institutional ethics committee. The metropolitan city of Delhi is divided into nine districts.(8) For the purpose of the present study, two districts of Delhi – South Delhi and South West Delhi – were together considered as the South Delhi region.

All the schools and colleges in the South Delhi region were included in the sampling frame. A two-stage cluster sampling design was used to draw a representative sample, of students in classes 9–12 in schools and in the first 2 years of graduation in colleges. These classes were chosen as they correspond to the desired age group of 14- to 19-year-old students. The first stage was a random selection of three schools from the list of schools, using a table of random numbers for the purpose. Similarly, two colleges were randomly selected from the list of colleges. The second stage comprised random sampling of one class each from standards 9-12 in each selected school. In the selected colleges, two classes each were randomly chosen from the first and second years of graduation.

## Inclusion/Exclusion criteria

All the students in the selected classes, present on the day of the survey, were eligible to participate, allowing for anonymous and voluntary participation. At the time of data analysis, the forms of respondents who had stated their age to be either less than 14 years or more than 19 years were excluded from the analysis.

## Consent

A written permission and consent from the principals was obtained prior to conducting the study in their school/college. A written consent was also obtained directly from the subjects who were above the age of majority.

## Statistical analysis

A pretested, semi-open-ended, and self-administered questionnaire was used in the study. The information collected was converted into a computer-based spreadsheet. Statistical analysis comprised calculating proportions of tobacco users and cross-tabulation with the chi-square test (Fisher’s exact test being applied, wherever applicable). Multiple logistic regression was applied to analyze the relationship between tobacco use and various independent variables under study. Stepwise logistic regression was used with the forward method of entry of variables into the model.

## Results

The age of the respondents ranged from 14 to 19 years. Overall, among the 550 respondents, there were 369 (67.1%) males and 181 (32.9%) females. A large majority of the respondents were Hindus (492 or 89.6%). Three-fourths belonged to a nuclear family and the remaining were part of a joint family.

The students were asked to mention having ever seen any of six “role models” smoking cigarettes [Table 1]. Nearly 31.6% of the students (174) had seen their father smoke, boys being more than girls in number. A very small number had seen their mother ever smoking. A sibling had been seen smoking by 28 (5.1%) and a best friend by 90 (16.4%). Large numbers of the respondents reported having seen their favorite celebrity smoking cigarettes (239 or 43.5%).

**Table 1:** ‘Role models’ ever seen smoking cigarettes by the students

Respondents having seen the role	Role models seen smoking					
model smoke	Father	Mother	Brother/sister	Best friend	Favorite teacher	Favorite celebrity
Male (*n*=369)	132	2	23	83	52	143
Percentage among males	35.8	0.5	6.2	22.5	14.1	38.8
Females (*n*=181)	42	0	5	7	1	96
Percentage among females	23.2	0	2.8	3.9	0.6	53

Having ever tried cigarette or bidi smoking was acknowledged by 88 (16.0%). Thirty-five students (about 40% of ever smokers) had first smoked a whole cigarette/bidi at an age of ≤13 years. A total of 39 (7.1%) students admitted being current smokers, i.e., they had smoked ≥1 time in past 30 days. Exactly 10% students (55) had ever tried smokeless forms of tobacco (like chewing tobacco, gutka, khaini, zarda, ‘paan’ with tobacco, etc.). With the exception of one female, the current usage of smokeless tobacco was exclusively restricted to male students [Table 2]. The prevalence of tobacco use overall, including smoking and smokeless form consumption, was found to be 20.9% in our study population.

**Table 2:** Health risk behaviors concerning tobacco use among the respondents

Behavior	Males		Females		Total	
	(*n*=369)		(*n*=181)		(*n*=550)	
	n	%	n	%	n	%
Ever tried cigarette or bidi	68	18.4	20	11.0	88	16.0
smoking						
Age at first smoking a whole	27	7.3	8	4.4	35	6.4
cigarette or bidi ≤ 13 years						
Current cigarette/bidi use	34	9.2	5	2.8	39	7.1
(≥1 time in past 30 days)						
Current heavy smoking	6	1.6	0	0.0	6	1.1
(≥5 per day on days of smoking)						
Ever tried smokeless tobacco	48	13.0	7	3.9	55	10.0
forms						
Current smokeless tobacco use	38	10.3	1	0.6	39	7.1
(≥1 time in past 30 days)						

The association of tobacco use (to mean ‘ever tobacco use’ henceforth in the article) with a range of sociodemographic factors of the adolescents was explored as part of the study. Tobacco use was higher among the students aged 16–19 than those in the age group of 14–15 years. Tobacco use was appreciably higher among the males (88; 23.8%) than the female respondents (27; 14.9%), (*P*=0.016). Tobacco use was higher among students from nuclear families (95; 23.3%) than joint families (20; 14.0%) (*P*=0.018).

Tobacco use was found to be considerably higher among students who had seen their brother/sister or their best friend smoke. The association with the mother’s smoking status could not be analyzed meaningfully because only two students reported having seen their mother ever smoke. Tobacco use was higher among students who had seen their favorite teacher smoke. The strong association of tobacco use by the subject with the number of role models ever seen smoking is exemplified in Table 3. Nearly half of the students who had seen three or more of the six role models smoking were found to be using tobacco themselves.

**Table 3:** Association of tobacco use behavior of the students with the number of role models ever seen smoking

Role models ever seen smoking	Number of	Tobacco use by
(out of six asked about)	respondents	the students
		*N* (%)
0	170	26 (15.3)
1-2	334	67 (20.1)
3 or more	46	22 (47.8)
Total	550	115 (20.9)

χ^2^=23.5, df=2, *P*=<0.001

Multivariate logistic regression was applied to explore the association of the tobacco use behavior with various socio-demographic variables. As depicted in Table 4, tobacco use was five times more likely if students had seen their sibling ever smoke (odds ratio 5.15, 95% confidence interval: 2.16–12.26), three times more likely if a best friend smoked (OR 2.92, 95% CI: 1.72–4.94), and two times more likely in students belonging to nuclear families (OR 1.96, 95% CI: 1.11–3.45).

**Table 4:** Significant correlates of “ever tobacco use” by the students on multivariate logistic regression analysis

Significant correlates	Category	Adjusted odds	*P* value
		ratio	
		(95% CI)	
Seen brother/sister smoking	No	1	<0.001
	Yes	5.15 (2.16–12.26)	
Seen best friend smoking	No	1	<0.001
	Yes	2.92 (1.72–4.94)	
Type of family	Joint	1	0.02
	Nuclear	1.96 (1.11–3.45)	

Only the correlates found to be significant at P <0.05 in the regression model are listed.

## Discussion

Health risk behaviors are an important link to the overall health status of adolescents and are important determinants of morbidity and mortality in this age group.(9) Nearly one-third of the students had seen their father smoking while a sibling had been seen smoking by 28 (5.1%). These are worrisome figures as the behaviors of the immediate family members are likely to influence the activities of the adolescents. Ninety students (16.4%) mentioned having seen their best friend smoke.

It is a matter of concern that among the boys, 52 (14.6%) reported having seen their favorite teacher smoke. Teacher’s use of tobacco has been found to be associated with the current use of tobacco among students.(10) An interesting finding was the high percentage of students who had seen their favorite celebrity smoking (39% boys, 53% girls). This could be explained by the fact that one particular movie actor very popular among adolescents was a known active smoker both off-screen and on-screen. Personalities in the public domain should realize that their resorting to the use of these substances in the public eye can seem like a virtual endorsement of these risk behaviors for their adolescent fans. A recent study by Sargent *et al*. (2007) too found a strong association between exposure to movie smoking and smoking by adolescents.(11)

Kotwal *et al*. (2005) in their study in the same region, that is South Delhi, found lifetime ever smoking by 18% of the school students in the age group of 15–17 years, a finding that closely matches ours.(12) Tobacco ever use among girls in our study (11.0%) however is much higher than the global average (6.0%) among girls of the age group of 15-19 years found in 1995.(13) The inference is that smoking rates are increasing rapidly among girls especially those in urban areas, a finding that supports the assertion in a WHO review.(3)

The present study found that tobacco use was significantly more among students who had seen their brother/sister (OR 5.2) or their best friend smoke (OR 2.9). Our findings are in agreement with the observation by WHO that adolescents whose parents or siblings smoke or whose friends do so are particularly likely to use tobacco themselves.(14) Jha (1994) too reported that examples set by siblings and parents, and peer pressure are strongly associated with tobacco use by young people.(15) Other studies in India support the observation too.(10,16) The results of a longitudinal study too emphasized that close friends’, siblings’, and parents’ smoking were all important influences on children’s smoking.(17)

The present study differs from the previous research works in the finding that a sibling seen smoking was found to have a stronger effect on tobacco use by the adolescent than peer influence. This could be explained by the fact that in the context of Indian culture, the family still retains a considerable influence over the adolescent as compared to western countries, where the adolescents seek to gain independence quite early and relate more with their peer group. Another factor could be that in the present study, only the smoking status of the best friend had been asked for, not of friends in general. Parental smoking has been found to be a strong predictor in the western studies. But, a father’s smoking influence was not found statistically significant in the present study and meaningful analysis of maternal smoking influence was precluded by the small number of mothers who had been seen smoking (only two). This study found a robust association of tobacco use by the subject with the number of role models ever seen smoking, quite similar to the findings of Taylor *et al*.(18)

A limitation of the present study is that the findings and their interpretations are restricted to adolescent students only. Further studies are needed that cover the groups of adolescents who are out of school or college, as the prevalence of health risk behaviors is likely to be higher among such adolescents. Also, detailed analysis of the social influence models for adolescent tobacco use was limited by the study being a cross-sectional one. Qualitative research methods like focused group discussions can be utilized in further studies to have in-depth analysis of the reasons for tobacco use among adolescent students.

## Conclusions

The findings in the study suggest that tobacco use is still an important risk behavior among adolescent students. This study found a strong association of tobacco use by the adolescents with having seen various role models ever smoking. Tobacco use cessation programs targeting the adolescents would need to be heedful of the influence of the role models on their target population also.

Regarding tobacco uses among students, stern steps need to be taken. Use in the campus should be strictly prohibited. Teachers should be motivated to not set any bad example by using tobacco in front of the students. IEC activities are needed to promote healthy behavior in the community because the behavior of various role models in their environment was seen to significantly influence the adolescents’ own risk status.
